# Prenatal paternal anxiety symptoms predict child DHEA levels and internalizing symptoms during adrenarche

**DOI:** 10.3389/fnbeh.2023.1217846

**Published:** 2024-01-04

**Authors:** Sherri Lee Jones, Victoria De Braga, Christina Caccese, Jimin Lew, Guillaume Elgbeili, Natalie Castellanos-Ryan, Sophie Parent, Gina Muckle, Catherine M. Herba, William D. Fraser, Simon Ducharme, Julia Barnwell, Jacquetta Trasler, Jean R. Séguin, Tuong-Vi Nguyen, Tina C. Montreuil

**Affiliations:** ^1^Department of Psychiatry, Faculty of Medicine and Health Sciences, McGill University, Montreal, QC, Canada; ^2^Department of Psychiatry, Research Institute of the McGill University Health Center, Montreal, QC, Canada; ^3^Department of Psychiatry, Douglas Research Center, Douglas Mental Health University Institute, Montreal, QC, Canada; ^4^School of Medicine, McGill University, Montreal, QC, Canada; ^5^Integrated Program in Neuroscience, McGill University, Montreal, QC, Canada; ^6^School of Psychoeducation, Université de Montréal, Montreal, QC, Canada; ^7^Centre de Recherche du Centre Hospitalier Universitaire (CHU) de Québec, School of Psychology, Laval University, Québec, QC, Canada; ^8^Centre Hospitalier Universitaire (CHU) Ste-Justine Research Centre, Université de Montréal, Montreal, QC, Canada; ^9^Department of Psychology, Université du Québec à Montréal (UQAM), Montreal, QC, Canada; ^10^Department of Obstetrics and Gynecology, Centre de Recherche du CHU de Sherbrooke, University of Sherbrooke, Sherbrooke, QC, Canada; ^11^Department of Pediatrics, Faculty of Medicine and Health Sciences, McGill University, Montreal, QC, Canada; ^12^Human Genetics and Pharmacology and Therapeutics, Faculty of Medicine and Health Sciences, McGill University, Montreal, QC, Canada; ^13^Department of Psychiatry and Addiction, Université de Montréal, Montreal, QC, Canada; ^14^Reproductive Psychiatry Program, McGill University Health Centre, Departments of Psychiatry and Obstetrics and Gynecology, Montreal, QC, Canada; ^15^Department of Educational and Counselling Psychology, McGill University, Montreal, QC, Canada

**Keywords:** paternal mental health, child development, DHEA, pituitary, MRI, internalizing, neuroendocrine, middle childhood

## Abstract

**Introduction:**

This study examined (1) whether measures of paternal anxious and depressive symptoms collected prenatally and during a follow-up assessment when the child was in middle childhood, predict child neuroendocrine outcomes, and (2) whether neuroendocrine outcomes are intermediate factors between paternal mental health and child cognitive/behavioral outcomes. Middle childhood coincides with increased autonomy as the child transitions into grade school, and with adrenarche, as the maturing adrenal gland increases secretion of dehydroepiandrosterone (DHEA) and its sulfated metabolite (DHEA-S), hormones that are implicated in corticolimbic development which regulate emotions and cognition.

**Methods:**

Participants were recruited from a subsample of a large prospective birth cohort study (3D study). We conducted a follow-up study when children were 6–8 years old (*N* = 61 families, 36 boys, 25 girls). Parental symptoms of anxiety, stress and depression were assessed via validated self-report questionnaires: prenatally using an in-house anxiety questionnaire, the Perceived Stress Scale (PSS) and the Center for Epidemiologic Studies Depression (CES-D), and at the follow up, using the Beck Anxiety and Beck Depression Inventories. Children provided salivary hormone samples, and their pituitary gland volume was measured from structural Magnetic Resonance Imaging (MRI) scans. Child behaviors were measured using the Strengths and Difficulties Questionnaire and cognitive outcomes using the WISC-V. Multiple regression analyses were used to test whether paternal mental health symptoms assessed prenatally and during childhood are associated with child neuroendocrine outcomes, adjusting for maternal mental health and child sex. Indirect-effect models assessed whether neuroendocrine factors are important intermediates that link paternal mental health and cognitive/behavioral outcomes.

**Results:**

(1) Fathers’ prenatal anxiety symptoms predicted lower DHEA levels in the children, but not pituitary volume. (2) Higher prenatal paternal anxiety symptoms predicted higher child internalizing symptoms via an indirect pathway of lower child DHEA. No associations were detected between paternal anxiety symptoms measured in childhood, and neuroendocrine outcomes. No child sex differences were detected on any measure.

**Conclusion:**

These results highlight the often-overlooked role of paternal factors during pregnancy on child development, suggesting that paternal prenatal anxiety symptoms are associated with child neuroendocrine function and in turn internalizing symptoms that manifest at least up to middle childhood.

## 1 Introduction

Parental factors, such as mental health, stress exposures, advanced age, socioeconomic status, and cognitive performance are associated with child neuroendocrine development. While it is well accepted that early-life exposure to maternal stress alters the programming of the developing neuroendocrine axes leading to lasting cognitive, behavioral, and physical effects in the offspring in childhood and into adulthood (Bergman et al., 2007, 2010; DiPietro, 2012; O’Donnell et al., 2013; Duchesne et al., 2017; Nguyen et al., 2018) the role of paternal factors has received much less attention. Paternal mental health symptoms including perceived stress (Challacombe et al., 2023), paternal anxiety (Möller et al., 2015) and paternal depression (Ramchandani et al., 2005; Wanless et al., 2008; Paulson et al., 2009; Gentile and Fusco, 2017; Cui et al., 2020), during the prenatal period and in childhood, are associated with child social, cognitive and behavioral outcomes. One possible link between paternal mental health and child outcomes could be via biological mechanisms including the child’s own neuroendocrine systems.

The prenatal period is critical in the structural development of neuroendocrine systems which develop in a sex-specific manner under the influence of steroid hormones, as shown in a classic and paradigm shifting study in rodents (Phoenix et al., 1959). That study proposed what is known as the organizational-activational hypothesis which is well-supported to this day and integrated into new conceptual frameworks of sexual differentiation (Arnold and Cahill, 2016). In brief, the organizational-activational hypothesis posits that prenatal testosterone released from the fetal testes (due to the testes-determining gene *Sry* located on the Y chromosome) (Goodfellow and Lovell-Badge, 1993) is critical for the structural organization of the sexually-dimorphic nucleus of the preoptic area of the hypothalamus resulting in a larger structure in males than in females. At puberty, gonadal steroid hormones activate those prenatally organized structures to elicit sex-specific hormone-dependent behaviors. Factors that alter the prenatal steroid hormone environment can disrupt the structural organization and function of the offspring’s hypothalamic–pituitary–adrenal and gonadal axes (HPA and HPG, respectively) (Glover et al., 2010; Gerecke et al., 2012; Jones et al., 2020). The consequence of this is altered behavioral expression that is dependent on the activation of those systems as shown in animal models (Ng, 2000; Welberg and Seckl, 2001; Austin et al., 2005; Kapoor and Matthews, 2008; Xiong and Zhang, 2013; Jones et al., 2015; McGowan and Matthews, 2017; Matthews and McGowan, 2019). Human observational studies suggest that prenatal factors such as parental stress may disrupt the prenatal steroid hormone environment resulting in altered physical and cognitive-behavioral outcomes in the offspring during childhood (Barrett et al., 2013, 2014).

Animal and human studies suggest that parental preconception and prenatal stress/mental health symptoms are associated with child neuroendocrine development and mental health outcomes (for reviews see, Glover et al., 2010; Chan et al., 2018; Tan et al., 2023). These associations may occur through maternal or paternal pathways, or some combination of both. For instance, during the preconception and prenatal periods, paternal stress or glucocorticoid administration in rodents (Dietz et al., 2011; Rodgers et al., 2013; Gapp et al., 2014; Short et al., 2016) or mental health problems (e.g., anxiety, depression, post-traumatic stress disorder in humans; Yehuda et al., 2014; Ayano et al., 2021) are associated with numerous offspring outcomes. Outcomes include higher anxiety symptoms in offspring that persist into adulthood (Ayano et al., 2021), disrupted epigenetic regulation of the glucocorticoid receptor gene in humans (Yehuda et al., 2014), impaired neurodevelopment in animals and humans (Chan et al., 2018), and altered steroid hormone levels in animals (Moisiadis et al., 2017) and humans (Black et al., 2019), as well as associations between steroid hormones and childhood internalizing problems as shown in humans (Black et al., 2019). In fact, paternal mental health status has been shown to alter sperm epigenetics, in turn influencing development of the offspring (Marcho et al., 2020). This may occur via paternal sperm epigenetics, and placental development (Crespi, 2020; Dini et al., 2021). Thus, paternal factors may play a role in child neuroendocrine and cognitive-behavioral development via sperm epigenetics or placental gene expression.

After birth, paternal psychosocial factors (e.g., parenting, perceived stress, the quality of the father-child relationship and engagement with childcare; De Cock et al., 2017; Challacombe et al., 2023; Wu et al., 2023) may also influence the child. Alternatively, associations between paternal stress or mental health and child development may also occur through maternal mental health, maternal stress, or the couple’s relationship quality. It has previously been shown that maternal stress during pregnancy disrupts typical associations between steroid hormones that are regulated by the HPA and HPG axes (Nguyen et al., 2018) and predicts earlier menarche in offspring (Duchesne et al., 2017). As such, paternal factors may interact with those of the mother, for example via increased stress levels, in turn predicting disrupted neuroendocrine development and function in the offspring.

A key developmental period for testing associations between paternal factors and the child’s neuroendocrine and behavioral development may become particularly important during middle childhood. During this time, children begin puberty while also gaining further independence from the parents, as they transition into elementary school. School transition is associated with increased morning salivary cortisol, suggesting it is a normative environmental stressor that activates the HPA axis (Parent et al., 2019; Leblond et al., 2022). For children who do not show an adaptive cortisol recovery, lower morning cortisol predicts higher anxiety symptoms 1.5 years later (Leblond et al., 2023), which supports the importance of considering associations between the HPA axis and mental health symptoms in childhood. Adrenarche, the first phase of puberty which begins around 6–8 years of age, is a neuroendocrine event, unique to humans and the great apes, characterized by enhanced secretion of adrenal hormones that are important for cognitive and behavioral development (Campbell, 2006). During this period, hypothalamic secretion of corticotropin-releasing factor (CRF) stimulates adrenocorticotropic hormone (ACTH) release from the anterior pituitary gland (PG). This triggers dehydroepiandrosterone (DHEA) and its sulfate DHEA-S production in the zona reticularis of the adrenal glands (Endoh et al., 1996); and it is during adrenarche that DHEA and DHEA-S become the most abundant hormones in the body (Adams, 1985; Campbell, 2006). The blood brain barrier is highly permeable to DHEA, while its sulfated DHEA-S metabolite is actively transported out of the central nervous system (Nguyen, 2018; Greaves et al., 2019; Cumberland et al., 2021). Higher DHEA levels during childhood and adolescence have been associated with better attention, working memory, reading, and writing abilities through alterations of structural brain networks (Nguyen et al., 2013, 2016, 2017, 2018; Nguyen, 2018). On the other hand, disruptions in the timing or rate at which adrenarche proceeds in a particular child is associated with adverse mental health problems (e.g., internalizing, externalizing problems; Dorn et al., 2008). Any DHEA-related optimization of cognitive skills may come at an emotional cost, with lower levels of social and affective functioning, particularly in vulnerable children with externalizing disorders, as previously proposed (Nguyen et al., 2013, 2016, 2017, 2018; Nguyen, 2018).

Thus, paternal factors including paternal stress, anxiety, and depressive symptoms during the prenatal and postnatal childhood periods (including concurrent symptoms assessed during childhood) may be associated with child neuroendocrine, cognitive and behavioral development, which may be particularly evident during middle childhood. However, studies in support of this neuroendocrine intergenerational transmission are limited by cross-sectional and retrospective data or were not specifically designed to examine the associations between parental factors, including paternal mental health, and child neuroendocrine and cognitive-behavioral development. Moreover, most of these studies do not account for the more specific roles of paternal mental health measured prenatally and into childhood.

Using data from a longitudinal birth cohort, the Design, Develop, and Discover (3D) Study (Fraser et al., 2016), we designed a follow-up study to examine whether paternal mental health symptoms assessed during the prenatal period and during childhood are associated with altered development of neuroendocrine structure (PG volume) and function (DHEA/DHEA-S levels) in middle childhood (6–8 years old), while controlling for maternal mental health. We hypothesized that paternal mental health symptoms would be associated with neuroendocrine structure (PG volume) and function (DHEA/DHEA-S levels) during adrenarche, though the predicted direction of those associations was unclear. We also hypothesized that the identified neuroendocrine factors would act as intermediates linking paternal mental health and child cognitive and behavioral development, given the role of adrenarche hormones on child brain and cognitive-behavioral development. Finally, we hypothesized that any associations between paternal mental health and child neuroendocrine outcomes would be more strongly predicted by prenatal mental health symptoms than symptoms assessed concurrently at the childhood assessment, given that neuroendocrine structural development is particularly sensitive to the prenatal period (Xiong and Zhang, 2013; Arnold and Cahill, 2016).

## 2 Materials and methods

### 2.1 Participants

Participants were recruited from the 3D (Design, Develop, Discover) Study, a prospective longitudinal birth cohort study (Fraser et al., 2016), initiated by the Integrated Research Network in Perinatology of Quebec and Eastern Ontario (IRNPQEO). Women were recruited during their first trimester of pregnancy, from university hospital centers in Québec, Canada, from June 2010 to September 2012. The 3D study included a total of 2,366 families. In the context of the present study, assessments include parental mental health (initial data collection of self-reported depressive, anxious, and stress symptoms, collected during the pregnancy) and parental highest level of education, measured during pregnancy.

We conducted an ancillary study examining the influence of paternal factors on child neuroendocrine development when children were 6–8 years of age. We focused on children born from spontaneous pregnancies (i.e., we did not include families that used assisted reproductive techniques) and mother–father-child trios who agreed to complete the 3D-transition follow-up study at 6 years old (Rioux et al., 2021) and to participate in additional data collection for our paternal study (Jones et al., 2023b). The 3D-transition study followed children as they transitioned from kindergarten to first grade, and involved extensive follow-ups (6 assessments, twice a year over 3 years). Of the 1,551, 3D study, families who agreed to be contacted for follow up, 939 participated in the transition study.

From the pool of 3D-transition study participants, the total sample size for our ancillary paternal-focused study (Jones et al., 2023b) included 61 families (36 boys, and 25 girls, biological sex determined at birth) ranging from 5.57 to 8.41 years old (girls, 5.57–8.41; boys, 6.01–8.10) and their parents. Our inclusion criteria comprised children with complete biosample profiles (e.g., placenta, umbilical cord blood, two-year-old blood samples, and paternal blood samples, for analyses outside the scope of the current study), and residing in the Greater Montreal area. Our exclusion criteria were children whose mothers reported smoking during the pre-conception period or during pregnancy, or whose fathers reported smoking during pregnancy, and children with abnormalities detected during physical exam indicative of an underlying medical condition (e.g., Prader-Willi or Turner syndromes, and neurological disorders affecting brain function).

The study was carried out in accordance with the Code of Ethics of the World Medical Association, and the axillary study data collected at 6–8 years old was approved by the Research Institute of the McGill University Health Center Research Ethics Board and the CHU Ste-Justine Research Center Ethics Board and conformed to the Declaration of Helsinki’s standards. All parents provided written informed consent and children provided verbal assent.

### 2.2 Procedure for paternal study: assessment at 6–8 years old

A registered nurse or trained research assistant, blind to the perinatal parental health factors, collected a medical history, the child’s vitals, height, weight, and anthropometric measurements (e.g., skinfold). The attending parent then completed a series of questionnaires, including the Puberty Development Scale (Robertson et al., 1992), the Beck Anxiety Inventory (BAI) and Beck Depression Inventory (BDI), as well as the Strengths and Difficulties Questionnaire (SDQ). Children then provided baseline saliva samples. The family was then brought to a 10-min mock MRI scanner to habituate the child with the aim of reducing potential movement artifacts in the official images. Next, the child underwent structural MRI, followed by saliva sample collections. Next, a clinical psychologist administered seven subtests of the Wechsler Intelligence Scale for Children (WISC-V), followed by the final saliva collections.

### 2.3 Measures

#### 2.3.1 Parental anxiety and depression scales

Prenatal measurements of perceived stress, anxiety and depressive symptoms were collected from 3D study data from both parents during the first or second trimester, using three self-report questionnaires: the Center for Epidemiological Studies Depression Scale (CES-D), the Perceived Stress Scale (PSS), and an Anxiety Scale (STR). Depressive symptoms experienced in the week prior were measured during the first trimester using the 10 item CES-D for mothers, and the 4-item version for fathers (Roberts and Vernon, 1983; Eaton et al., 2004). The 4-item version has been shown to be highly correlated with longer versions of CES-D (Melchior et al., 1993; Zauszniewski and Graham, 2009). Scores range from 0 to 30 (maternal) or 0–12 (paternal), with higher scores denoting greater depressive symptoms. Cronbach’s α in the current sample was 0.81 for mothers and 0.61 for fathers CESD. These reliability measures are consistent with the literature. Lower reliability in fathers versus mothers has been reported by others (Schudlich and Cummings, 2007) and reliability decreases with fewer items (Carpenter et al., 1998). Stress and anxiety symptoms were measured using two questionnaires. The 4-item version of the Perceived Stress Scale (PSS) completed during the first trimester, measures nonspecific perceived stress in the previous month, with higher scores indicating a greater perception of stress (Warttig et al., 2013). The anxiety-screening questionnaire (STR), designed by the 3D study principal investigators, and completed by fathers during the first trimester visit, and by mothers during the second trimester visit, was developed for use in large scale longitudinal studies. It uses 10 items to screen for 10 anxiety disorders based on DSM-IV criteria (Shapiro et al., 2017). In the current sample, Cronbach’s α = 0.78 for maternal STR and 0.85 for paternal STR; and α = 0.86 for maternal PSS, and 0.71 for paternal PSS.

At the 6–8-year-old assessment, parental anxiety and depressive symptoms (termed concurrent mental health symptoms in the present manuscript) were assessed using the 21-item versions of the BAI and BDI. The BAI measures the severity of anxiety symptoms with high test–retest reliability and internal consistency, with minimal scores ranging from 0 to 7, mild scores ranging from 8 to 15, moderate scores ranging from 15 to 25, and scores 26 and above rated as severe, indicating concerning levels of anxiety (Beck et al., 1988a; Creamer et al., 1995). The BDI reliably measures symptoms of depression with high internal consistency; scores ranging from 0 to 13 indicate minimal depressive symptoms, 14–18 mild to moderate, and 19–29 as moderate to severe, and scores above 30 alluding to severe depression (Beck and Steer, 1984; Beck et al., 1988b). Within the current sample, Cronbach’s α = 0.92 for mothers and α = 0.85 for fathers on the BDI, and Cronbach’s α = 0.88 for mothers and α = 0.791 for fathers on the BAI.

#### 2.3.2 Child behavioral scales

##### 2.3.2.1 Strengths and difficulties questionnaire

The Strengths and Difficulties Questionnaire (SDQ) (Goodman, 1997) is an emotional and behavioral screening questionnaire, consisting of 25 items that comprise 5 subscales (emotional, peer, conduct, hyperactivity and prosocial) that each range from 0 to 10 points. An internalizing variable was computed by summing the emotional and peer subscales, and an externalizing variable was computed by summing the conduct and hyperactivity subscales (Goodman et al., 2010). Behavioral analyses were conducted on the internalizing and externalizing variables, as well as the prosocial subscale. If the internalizing or externalizing analyses were statistically significant, the analyses were conducted separately on their respective subscales to help identify which particular behavioral symptoms are driving the associations. In the current sample, Cronbach’s alpha for the prosocial subscale was 0.69 for the internalizing subscale it was =0.66, and for the externalizing subscale it was =0.86.

##### 2.3.2.2 Wechsler intelligence scale for children – fifth edition

A trained psychologist administered seven subtests of the WISC-V to participating children (i.e., Block Design, Similarities, Matrix Reasoning, Digit Span, Coding, Vocabulary and Figure Weights). Full scale IQ was computed from all subtests. A Verbal Comprehension measure was created by summing the scores on Similarities and Vocabulary and Figure Weights, whereas an index of Fluid Reasoning was created by summing scores on Matrix Reasoning and Figure Weights. WISC-V has been shown to be reliable (all subtests range from α = 0.80 to 0.94) and valid (factor analysis showing WISC-V primary subtests to be associated with different aspects of cognitive ability) for children aged 6–16 years old, and its clinical relevance is supported through its association with Child and Adolescent Academic Questionnaire (containing items related to risk factors for school failure; *r* = −0.50) and Child and Adolescent Behavior Questionnaire (containing items related to risk factors for delinquency and criminal behavior; *r* = −0.12) (Pearson, 2017). Cronbach’s alpha in the current study was 0.80 for the full scale WISC.

#### 2.3.3 Saliva collection and assaying

Saliva samples were collected at three time points: baseline (pre-), post-, and 1-h-post MRI, using a salivary swab (Sarstedt Salivettes, #51.1534.500), for a total of six samples; only the baseline samples were used in the current study. Two consecutive samples were taken at each time point to get sufficient sample to measure multiple analytes. Each sample was collected for a one-minute period. DHEA was measured before DHEA-S, resulting in fewer available samples for DHEA-S analysis. The time (hh:mm) of each sample collection was also documented and accounted for as a covariate in the statistical analyses. After collection, samples were centrifuged and frozen at −20°C until assayed. The supernatant was assayed for salivary hormone levels including DHEA and DHEA-S, using Salimetrics Enzyme-Linked Immonosorbant Assay kits (DHEA: Salimetrics Cat# 1–2,212-5; intraCOV% = 1.7; interCOV% = 15.7, DHEA-S: Cat.#1–1,252, intraCOV% = 2.5; interCOV% = 15.3). The average participant salivary hormone levels from the two samples for each time point were then computed, calculating the mean if both values were available, or using the available value if one value was missing. Then, using the Expectation–Maximization method (Dong and Peng, 2013), the mean values from hormones of all time points were imputed, adjusting for child sex at birth, season of saliva sample collection, paternal and maternal ethnicity (Caucasian [*n* = 48] vs. non- Caucasian [*n* = 13]), gestational age at birth, arm and back skinfold measures, lean body mass, BMI percentile, age of child at the time of assessment, as well as time of day of first saliva collection. These variables were selected based on known associations with hormone data. Participants that had two missing values at any one of the three collection time points for a given hormone (DHEA, *n* = 3; DHEA-S, *n* = 24) were assigned as missing values (e.g., a participant missing all DHEA values at each timepoint before imputation would not have any after imputation either).

#### 2.3.4 MRI

##### 2.3.4.1 MRI acquisition

Children were scanned using a Philips Achieva 3 T MRI and 32 channel head coil, and structural T1- and T2-weighted images were acquired, (note that NODDI MRI scans were also acquired, for analyses that are outside the scope of the current study). The MRI technician evaluated the acquired image quality, and if deemed unclear, the scan was repeated. Four children did not complete the MRI scans.

For the purpose of this project, only the T1- and T2-weighted images were used. T1-weighted 3D Turbo Field Echo (TFE) scans were acquired in the sagittal plane TR/TE 8.2/3.7 ms; flip angle 8 degrees, 180 slices, resolution 240, FoV = 240 × 240 × 180, 1 mm isotropic, with a total scan duration of 6 m 20 s per subject. T2-weighted 3D images were acquired using a Turbo Spin Echo (TSE) sequence, in the sagittal plane, TR/TE = 2500/234 ms, flip angle 90°, 1 mm isotropic resolution, FoV: 240 × 240 × 180, 180 slices. T2 scan time was 5 m 32 s.

##### 2.3.4.2 Image processing

T1- and T2-weighted DICOM images were converted to MINC format (Montreal Neurological Institute).^1^ Inspection of a subset of .jpg images created from selected axial, coronal, and sagittal planes of the scans provided a rapid visual confirmation of the conversion process (e.g., scan orientation and identification). Two raters (VB and SLJ) assessed the quality of the scans around the pituitary gland to ensure the images were clear enough for manual segmentation on the T1W images. One scan was assessed by both raters as poor quality; however, the scan was included in the analyses because the manual pituitary segmentation protocol was still easily applied, and this participant was not an outlier on the pituitary volumetric measures. All other images passed the quality control assessment and were included in the analyses.

Brain images were then preprocessed using the open-source MINC Toolkit (version 1.9.10) prior to segmentation. First, for each subject, the T2-weighted image was rigidly aligned to the T1-weighted image using *mritoself*. Next, the T1- and aligned T2-weighted images were corrected for magnetic field non-uniformities (Sled et al., 1998), linearly registered to standard stereotaxic space and resampled onto a 0.5 mm voxel grid (ICBM152b) and intensity normalized (Collins et al., 1994).

##### 2.3.4.3 Manual MRI image segmentation

The image segmentation software, Display 2.0, developed at the Montreal Neurological Institute (MINC Tool Kit and Display) was used to manually segment the PG from 57 available processed T1-weighted images, with cross-checking the T2-weighted image. All segmentations were done blind to the sex at birth of the child and to the predictors.

**Figure 1:**
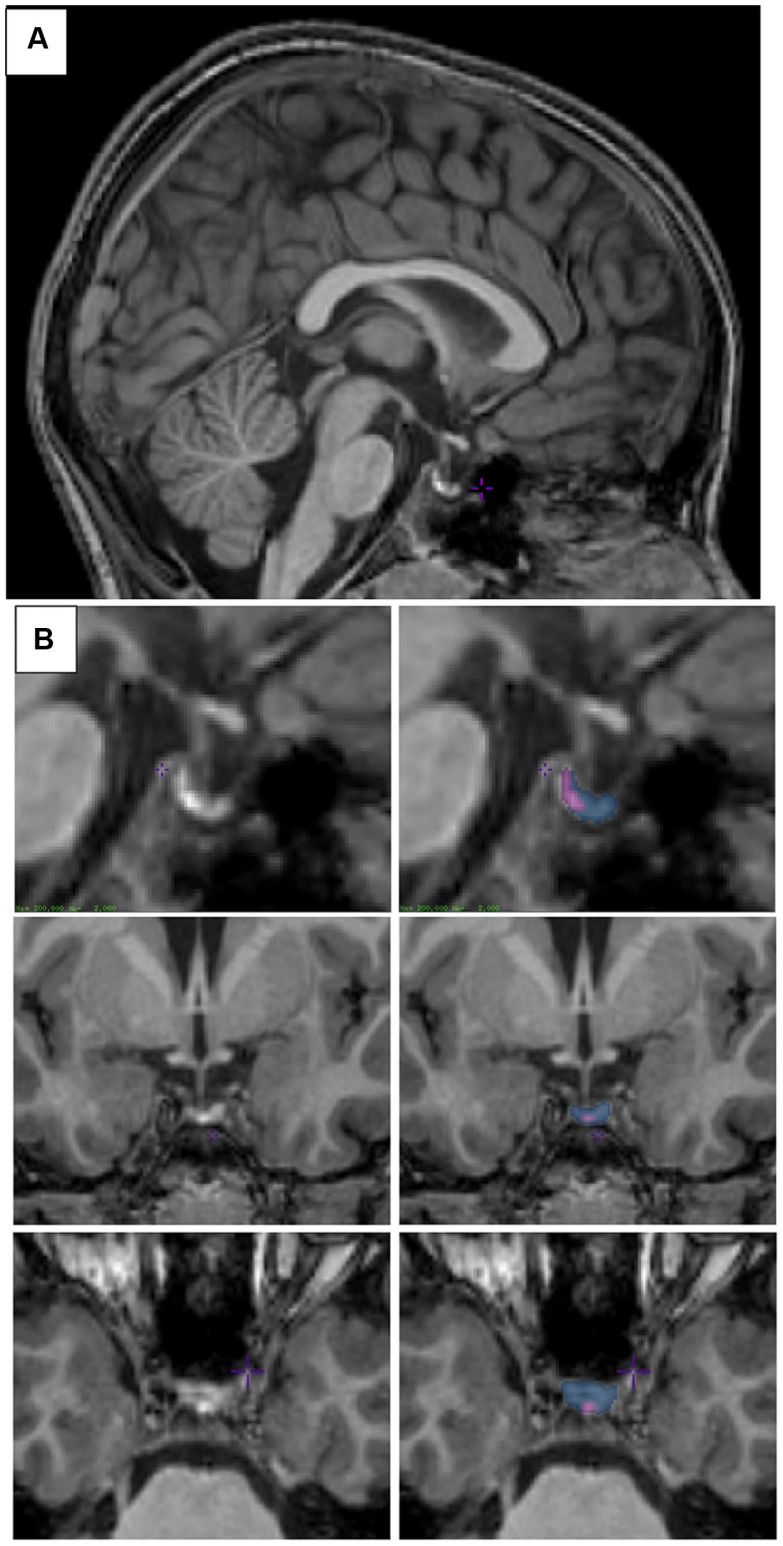
Pituitary segmentation. **(A)** Midsagittal view of the pituitary gland from a T1-weighted MRI image of a 6–8-year-old child. **(B)** Sagittal (top), coronal (middle), and axial (bottom) views of the pituitary gland from a T1-weighted MRI image. Left column shows unsegmented image, right column shows the posterior pituitary labeled in purple and the anterior pituitary labeled in blue. Images were viewed and segmented in Display (minctools, MNI).

The PG can easily be identified in the mid-sagittal plane, inferior to the hypothalamus (Figure 1A). It is bordered by the sphenoid sinus anteriorly and ventrally, the cavernous sinuses laterally, the dorsum sellae posteriorly, and the diaphragma sellae dorsally (Anastassiadis et al., 2019). Pituitary segmentation was performed as in Jones et al. (2023a). Visualization of the PG was maximized by adjusting the contrast such that each voxel within the PG showed variation in contrast before the start of segmentation. Manual segmentation of the PG began in the sagittal view, by outlining a hypointense signal located between the anterior and posterior lobes on the T1-weighted image. The identification of this signal used to delineate the anterior and posterior lobes was first drawn using the label assigned to the posterior PG on all sagittal sections where the line was clearly visible. Then, moving from superior to inferior, we segmented the posterior lobe in the axial view on all sections where the hypointense signal was clearly visible. Next, the most anterior extent of the anterior pituitary was determined using the sagittal view. In the coronal orientation, the superior, inferior, and lateral boundaries of the anterior PG were drawn to obtain the full segmentation. An example is shown in Figure 1B. Pituitary glands were segmented by two raters (VB and SLJ). Inter (between VB and SLJ) and intra- rater (VB) reliabilities were calculated from three randomly selected scans. The level of overlap between two labels was assessed using the Dice kappa (Ƙ) metric (Chakravarty et al., 2008), for inter- and intra-rater reliability:


$$Κ=\frac{2a}{\left(2a+b+c\right)}$$

Where *a* represents the number of common voxels between the two labels, and *b* and *c* represent the sum of the voxels uniquely identified by either label, respectively. Scores greater than 0.7 are deemed acceptable. Reliability for anterior and posterior segmentations were good (intra-rater Ƙ = 0.86 and 0.76; inter-rater Ƙ = 0.81 and 0.74, respectively).

PG volume (mm^3^) was extracted using the *print_all_labels* command available in the minc-toolkit, which provides a summation of all labeled voxels and then multiplied by the voxel size to obtain the final volume.

### 2.4 Statistical analyses

Analyses were conducted using SPSS (version 24) and the PROCESS macro (Hayes, 2013) (version 2.16.3) for the indirect-effect (mediation) analyses. Independent samples t-tests were used to test for child sex differences on each of the key variables. DHEA and DHEA-S were positively skewed (Shapiro–Wilks *p* < 0.05), however, log transformations did not improve normality (Shapiro–Wilks *p* < 0.05). Thus, we conducted the analyses using the raw, untransformed data. Bivariate Pearson correlations were used to assess associations between predictors, potential confounding variables and outcome variables. Multiple regression was used to examine whether paternal prenatal anxiety, stress, or depressive symptoms (as measured by the STR, PSS, and CES-D, respectively, in separate models) predicted PG volumes or baseline DHEA/DHEA-S while controlling for sex and the equivalent measure of maternal prenatal anxiety, stress or depressive symptoms. Next, we investigated whether paternal childhood (concurrent) anxiety or depressive symptoms (measured using the BAI or BDI, respectively) predicted PG volumes or baseline DHEA/DHEA-S, while controlling for sex and the equivalent measure of maternal mental health. Sex and maternal mental health factors were always included as covariates based on their extensive roles in child outcomes as documented in the literature (DiPietro, 2012; Glover and Hill, 2012; Kim et al., 2015; Janssen et al., 2016).

Two separate analyses were used to examine paternal-child relationships: (1) linear regression models adjusted for sex of the child, and the equivalent measure of maternal mental health (CES-D, PSS, STR for prenatal measures; BAI, BDI for concurrent measures); and (2) next, for those models that revealed significant associations between paternal mental health and child neuroendocrine outcomes, over and above maternal mental health, we conducted additional linear regression models adjusted for additional potential confounding variables (in separate models, to increase power of detecting potential roles of the confounding variables, while minimizing multicollinearity among paternal mental health indicators and a model over-fitting problem). Covariates were highest level of maternal education, highest level of paternal education, annual household income, season of hormone sampling, and time of day of hormone sampling. Observed power was computed using G*Power for MacOS (Version 3.1.9.6), and together with partial correlations (*r*_p_) (Ferguson, 2009), these metrics were used to interpret measured associations.

Next, for significant regression models predicting child PG or DHEA/DHEA-S from the paternal measure, indirect-effect models were conducted using mediation models in PROCESS, to examine whether those associations explained child behavioral outcomes as measured with the SDQ, and WISC-V controlling for sex and the equivalent maternal mental health measures. To examine the relative contribution of paternal anxiety or depressive symptoms assessed in the prenatal period and in middle childhood (no parental stress measures were taken in childhood), we ran two indirect-effects models that each included both the prenatal and middle childhood paternal mental health measure: the first model entered the prenatal paternal measure as the predictor (controlling for the childhood paternal measure) and the second model entered the middle childhood paternal measure as the predictor (controlling for the prenatal paternal measure). Because the same variables were included in both models, and only the prenatal/childhood paternal predictor was switched, this allowed us to compare the relative contribution of one versus the other. Significance levels for all analyses was set at *p* < 0.05. Indirect-effects analyses were considered significant when the confidence interval excluded 0.

## 3 Results

### 3.1 Sample characteristics

Sample characteristics are listed by sex and total sample in Table 1. All saliva samples were collected in late afternoon. Two boys had higher levels of DHEA-S that were considered extreme outliers on SPSS boxplots, indicating that their DHEA-S level was at least 3 times higher than the inter-quartile range. However, because they had normal DHEA levels, pituitary volume, parental mental health measures and behavioral outcomes relative to the rest of the sample, and because the DHEA-S levels were within the kit range, we included these participants in all analyses. Scatterplots of correlations (with and without the DHEA-S outliers) between hormone levels and paternal stress, anxiety, and depression measures are presented in Supplementary Figure S1 (DHEA) and Supplementary Figure S2 (DHEA-S).

**Table 1:** Sample characteristics of the paternal study sample.

	Boys		Girls		Total
	*N*	Mean (SD)	*N*	Mean (SD)	Mean (SD)
Child’s age at assessment (years)	36	6.68 (0.48)	25	6.64 (0.63)	6.66 (0.54)
Season of hormone sampling^a^	36	1.94 (1.01)	25	2.36 (0.21)	2.12 (1.03)
Time of day of first hormone sampling (hh:mm)	36	15:45 (0:44)	25	15:38 (0:45)	15:43 (0:44)
Mother highest level of education^b^ (at prenatal assessment)	36	6.75 (1.52)	25	6.90 (1.22)	6.81 (1.40)
Father highest level of education^b^ (at prenatal assessment)	36	6.64 (1.76)	25	6.52 (1.33)	6.59 (1.56)
Annual household income^c^ (at prenatal assessment)	36	7.25 (2.27)	25	7.44 (2.02)	7.33 (2.16)
**Paternal predictors [scale range]**					
*Prenatal assessment*					
CES-D [0–12]	35	0.83 (1.44)	23	0.83 (1.07)	0.83 (1.30)
PSS [0–12]	35	3.26 (2.65)	23	3.83 (2.84)	3.48 (2.72)
STR [0–44]	35	6.20 (6.23)	23	5.30 (4.72)	5.85 (5.65)
*Childhood assessment*					
BAI [0–63]	31	4.03 (3.92)	24	3.62 (4.76)	3.85 (4.27)
BDI [0–63]	31	3.82 (4.97)	24	4.04 (3.84)	3.91 (4.47)
**Maternal predictors [scale range]**					
*Prenatal assessment*					
CES-D [0–30]	34	7.55 (5.18)	21	8.33 (4.82)	7.85 (5.02)
PSS [0–12]	34	3.79 (3.01)	22	4.73 (3.07)	4.16 (3.04)
STR [0–44]	29	6.62 (4.92)	23	6.61 (5.51)	6.61 (5.13)
*Childhood assessment*					
BAI [0–63]	36	6.22 (7.70)	25	5.60 (4.93)	5.97 (6.67)
BDI [0–63]	36	6.67 (7.64)	25	7.96 (8.22)	7.20 (7.84)
**Child outcomes [scale ranges]**					
Baseline DHEA (pg/mL)	33	58.28 (67.28)	25	51.55 (57.42)	55.38 (62.78)
Baseline DHEA-S (pg/mL)	24	897.67 (2852.53)	13	212.49 (279.24)	656.93 (2309.67)
DHEA Imputed (pg/mL)	36	55.32 (65.33)	25	51.55 (57.42)	53.77 (61.74)
DHEA-S Imputed (pg/mL)	36	684.00 (2346.10)	25	403.87 (612.18)	569.19 (1838.47)
Anterior pituitary volume (mm^3^)	34	347.21 (71.59)	23	385.06 (109.61)	362.48 (89.95)
Posterior pituitary volume (mm^3^)	34	89.89 (27.40)	23	80.39 (29.96)	86.05 (28.58)
Total pituitary volume (mm^3^)	34	437.10 (78.22)	23	465.45 (114.96)	448.54 (94.84)
*SDQ [0–10 for each subscale]*					
Internalizing [0–20]^d^	36	2.72 (2.32)	25	2.04 (2.52)	2.44 (2.41)
Externalizing [0–20]^e^	36	5.86 (4.54)	25	5.36 (4.17)	5.66 (4.37)
Emotional subscale	36	1.75 (1.95)	25	1.32 (1.84)	1.57 (1.90)
Peer subscale	36	0.97 (1.25)	25	0.72 (0.94)	0.87 (1.13)
Conduct subscale	36	2.00 (1.99)	25	1.52 (1.71)	1.80 (1.88)
Hyperactivity subscale	36	3.83 (3.01)	25	3.84 (2.98)	3.85 (2.97)
Prosocial subscale	36	8.61 (1.70)	25	8.64 (1.60)	8.62 (1.64)
*WISC-V*					
Full Scale	36	112.56 (13.73)	25	111.20 (11.75)	112.00 (12.87)
Verbal	36	112.72 (15.44)	25	113.76 (9.88)	113.15 (13.25)
Fluid reasoning	36	110.08 (11.79)	25	108.72 (13.87)	109.53 (12.59)

*N* = 61 families at the 6–8-year-old assessment. BAI, Beck Anxiety Inventory; BDI, Beck Depression Inventory; CES-D, Center for Epidemiological Studies Depression Scale; PSS, Perceived Stress Scale; STR, Anxiety Scale; SDQ, Strengths and Difficulties Questionnaire; WISC-V, Wechsler Intelligence Scale for Children – Fifth Edition. ^a^Season coded as 1: Spring, 2: Summer, 3: Fall, 4: Winter. ^b^Education coded as 0, no schooling; 2, completed elementary; 3, did not complete high school; 4, completed high school; 5, post-secondary education not completed; 6, CEGEP, college, technical training or nurse training; 7, completed university; 8, masters complete; 9, doctorate complete. ^c^Household income coded as: 0: <10,000, 1: 10000–19,999; 2: 20000–29,999; 3: 30000–39,999; 4: 40000–49,999; 5: 50000–59,999; 6: 60000–69,999; 7: 70000–79,999; 8: 80000–99,999; 9: ≥ 100,000. ^d^Internalizing is the sum of Emotional and Peer subscales; ^e^Externalizing is the sum of Conduct and Hyperactivity subscales. No sex differences were detected on any of the variables.

None of the key variables differed between sexes. Specifically, no sex differences were detected on any of the parental mental health measures, on child DHEA/DHEA-S levels, pituitary gland volume, nor on child SDQ or WISC scores, nor between child’s age on test day (all *p* > 0.12). Analyses were thus conducted on the full sample. Pearson correlations between paternal predictors, covariates, and child PG, behavioral and cognitive outcomes are displayed in Table 2.

**Table 2:** Correlation matrix between maternal and paternal predictors, child outcomes, and covariates.

	1	2	3	4	5	6	7	8	9	10	11	12	13	14	15	16	17	18	19	20	21	22
*Paternal predictors*																						
1. Paternal CES-D																						
2. Paternal PSS	0.670^**^																					
3. Paternal STR	0.663^**^	0.661^**^																				
4. Paternal BAI	0.250	0.314^*^	0.515^**^																			
5. Paternal BDI	0.483^**^	0.362^**^	0.484^**^	0.489^**^																		
*Control variables*																						
6. Maternal CES-D	0.204	0.261	0.202	0.096	−0.106																	
7. Maternal PSS	0.243	0.190	0.195	−0.017	−0.108	0.724^**^																
8. Maternal STR	0.072	0.124	0.263	0.328^*^	0.046	0.512^**^	0.314^*^															
9. Maternal BAI	0.013	0.133	0.162	0.503^**^	0.312^*^	0.094	0.089	0.265														
10. Maternal BDI	−0.045	0.077	0.074	0.432^**^	0.105	0.478^**^	0.440^**^	0.420^**^	0.598^**^													
11. Season of child hormone sampling	0.132	0.041	0.000	−0.062	0.047	0.083	0.089	0.102	0.061	−0.099												
*Child outcomes*																						
12. DHEA imputed (pg/mL)	−0.120	−0.078	−0.224	0.205	−0.138	0.020	0.132	0.178	−0.047	0.131	−0.114											
13. DHEA-S imputed (pg/mL)	0.100	−0.018	−0.094	0.117	−0.107	0.082	−0.045	−0.120	−0.062	−0.020	0.017	0.266^*^										
14. Anterior pituitary volume	0.157	0.163	0.022	0.128	−0.066	0.087	−0.014	0.023	−0.084	0.046	−0.218	0.083	0.384^**^									
15. Posterior pituitary volume	0.055	0.146	0.044	−0.114	0.050	0.059	−0.026	−0.027	0.078	−0.066	0.073	−0.125	0.017	0.017								
16. Total pituitary volume	0.165	0.197	0.034	0.088	−0.048	0.099	−0.021	0.012	−0.056	0.024	−0.185	0.041	0.370^**^	0.954^**^	0.318^*^							
17. SDQ internalizing	−0.114	0.038	0.024	0.182	0.164	0.320^*^	0.196	0.009	0.400^**^	0.445^**^	−0.074	−0.209	0.003	0.015	0.135	0.055						
18. SDQ externalizing	−0.231	−0.081	−0.045	0.049	−0.124	0.211	0.043	0.379^**^	0.244	0.369^**^	−0.094	0.027	−0.098	−0.063	−0.006	−0.061	0.326^*^					
19. SDQ conduct scale	−0.194	−0.065	−0.025	0.112	−0.083	0.190	0.028	0.311^*^	0.203	0.262^*^	−0.074	−0.018	−0.117	−0.034	0.024	−0.025	0.373^**^	0.839^**^				
20. SDQ emotional scale	−0.039	0.118	0.117	0.270^*^	0.277^*^	0.257	0.167	0.164	0.455^**^	0.452^**^	−0.009	−0.168	0.005	0.067	0.093	0.092	0.889^**^	0.279^*^	0.401^**^			
21. SDQ hyperactivity scale	−0.215	−0.077	−0.049	0.002	−0.130	0.188	0.045	0.360^**^	0.230	0.377^**^	−0.092	0.051	−0.069	−0.071	−0.025	−0.074	0.244	0.939^**^	0.601^**^	0.157		
22. SDQ peer scale	−0.176	−0.113	−0.140	−0.068	−0.118	0.252	0.139	−0.292^*^	0.088	0.189	−0.143	−0.165	−0.002	−0.081	0.133	−0.037	0.638^**^	0.227	0.121	0.214	0.257^*^	
23. SDQ prosocial scale	0.205	0.111	0.079	−0.151	0.144	−0.182	−0.098	−0.116	−0.097	−0.297^*^	0.026	0.017	0.139	0.076	0.122	0.109	−0.407^**^	−0.392^**^	−0.488^**^	−0.361^**^	−0.267^*^	−0.260^*^
24. WISC full scale	−0.072	−0.118	0.013	0.203	0.244	−0.037	0.091	−0.070	0.009	0.049	−0.053	0.113	−0.169	−0.022	−0.141	−0.063	0.119	−0.376**	−0.314*	0.128	−0.354**	0.039
25. WISC verbal	−0.073	−0.120	0.036	0.050	0.033	0.060	0.128	−0.033	−0.082	0.006	−0.007	−0.051	−0.008	0.025	0.021	0.030	0.140	−0.261*	−0.257*	0.031	−0.221	0.246
26. WISC fluid reasoning	0.002	−0.013	0.155	0.299*	0.305*	0.015	0.121	−0.105	0.086	0.144	−0.206	0.087	−0.250	−0.080	−0.226	−0.144	0.140	−0.284*	−0.194	0.182	−0.295*	−0.007

*N* = 46–61 depending on variable. BAI, Beck Anxiety Inventory; BDI, Beck Depression Inventory; CES-D, Center for Epidemiological Studies Depression Scale; PSS, Perceived Stress Scale; STR, Anxiety Scale; SDQ, Strengths and Difficulties Questionnaire. Statistically significant associations are shown in bold. ^*^p < 0.05, ^**^p < 0.01.

Paternal anxiety, stress, and depressive factors were moderately correlated (*r* = ~0.66 for prenatal measures; *r* ~ 0.4 for postnatal measures, all *p* < 0.01). DHEA-S was weakly correlated with total PG volume, which appears to be driven by anterior PG volume as DHEA-S was not correlated with posterior PG volume.

### 3.2 Prenatal paternal mental health, child pituitary volume and hormones of adrenarche

Linear regression models were used to examine whether prenatal paternal anxiety symptoms, perceived stress, or depressive symptoms measured by the STR, PSS or CES-D predict pituitary volume and DHEA/DHEA-S levels in the 6–8-year-old offspring, controlling for sex of the child and the equivalent maternal mental health factors. We found that higher paternal anxiety symptomatology during pregnancy (STR) was associated with lower DHEA levels in the child (*B* = −3.025, *SE* = 1.475, *β* = −0.293, *p* = 0.046, *r*_p_ = −0.287, observed power = 0.88), controlling for prenatal maternal STR and sex. Sex was not significant in the model (*B* = −3.688, *SE* = 16.711, *p* = 0.826, *r*_p_ = −0.032, observed power = 0.15). As shown in Table 3, the association between paternal STR and child DHEA remained significant when adjusting for each covariate entered one at a time in separate regression models (highest level of maternal and paternal education, season of hormone sampling, time of day of hormone sampling, and annual household income before taxes). We also conducted a sensitivity analysis, adding postnatal parental anxiety indicators (paternal and maternal BAI) to the model, and the association between paternal STR and child DHEA was maintained (*B* = −3.901, *SE* = 1.905, *β* = −0.355, *p* = 0.047, *r*_p_ = −0.312), whereas paternal anxiety symptoms measured in childhood was not a significant predictor in this model (*B* = 3.822, *SE* = 2.547, *p* = 0.142, *r*_p_ *= 0.234*). We note that paternal anxiety (assessed using the STR), as a predictor, had a consistent effect size, and the observed power was greater than 0.80, even after controlling for covariates; these metrics suggest that the sample size was sufficient to detect the association between paternal STR and child DHEA if the effect size is a good estimate of the true population effect size (Table 3). Sensitivity analyses were conducted by excluding the two participants with high DHEA-S values, and the associations were maintained, although slightly attenuated (*B* = −2.778, *SE* = 1.466, *β* = −0.274, *p* = 0.065, *r*_p_ = 0.272). In contrast, the data did not support our hypothesis that prenatal paternal mental health measures would be associated with pituitary volumes, given that no significant association was detected. We however note that the effect sizes for paternal mental health predicting pituitary volumes were small (*r*_p_ < 0.218) and the observed power were not large (0.180–0.787), suggesting that if these small effect sizes are reliable estimates of the true population effect sizes, then we were underpowered (Supplementary Table S1).

**Table 3:** Summary of multiple regression analyses for prenatal paternal anxiety (STR) predicting DHEA levels (imputed) adjusted for covariates in separate models.

	*R*^2^	Unstandardized B	SE of B	*β*	*r*_p_	*p*	Observed power
*STR to DHEA imputed*							
Maternal education	0.180						
Paternal STR		−3.032*	1.439	−0.293	−0.297	0.041	0.857
Maternal STR		3.913*	1.725	0.318	0.317	0.028	0.881
Sex^a^		0.045	16.432	0.000	0.000	0.998	0.050
Maternal education		−10.455	5.699	−0.250	−0.261	0.073	0.803
Paternal education	0.133						
Paternal STR		−3.030*	1.484	−0.293	−0.289	0.046	0.846
Maternal STR		3.646*	1.772	0.296	0.290	0.045	0.848
Sex^a^		−3.954	16.766	−0.033	−0.035	0.815	0.145
Paternal education		4.456	5.298	0.117	0.123	0.405	0.450
Season of hormone sampling	0.142						
Paternal STR		−2.975*	1.473	−0.288	−0.285	0.049	0.841
Maternal STR		3.647*	1.755	0.296	0.293	0.043	0.852
Sex^a^		1.143	17.266	0.099	0.010	0.948	0.074
Season of sampling^b^		−9.051	8.353	−0.154	−0.158	0.284	0.558
Time of hormone sampling	0.123						
Paternal STR		−3.057*	1.490	−0.296	−0.290	0.046	0.848
Maternal STR		3.522	1.778	0.286	0.280	0.054	0.833
Sex^a^		−3.110	16.916	−0.026	−0.027	0.855	0.121
Time of sampling		0.001	0.003	0.059	0.062	0.677	0.234
Annual household income	0.188						
Paternal STR		−3.386*	1.444	−0.328	−0.327	0.023	0.892
Maternal STR		3.123	1.702	0.254	0.261	0.073	0.803
Sex^a^		−1.617	16.259	−0.013	−0.15	0.921	0.534
Annual household income		−7.372	3.754	−0.266	−0.278	0.056	0.830

*N* = 51. ^*^p < 0.05. STR, anxiety scale; DHEA, dehydroepiandrosterone. ^a^0 = boy; 1 = girl. ^b^1 = Spring, 2 = Summer, 3 = Fall, 4 = Winter. *r*_p_ = partial correlation.

### 3.3 Paternal mental health measured in childhood, child pituitary volume, and hormones of adrenarche

We examined whether paternal mental health measured concurrently at the childhood assessment (BAI or BDI) was associated with DHEA/DHEA-S levels in their 6–8-year-old offspring, using linear regression models while controlling for the equivalent measure of maternal mental health and sex. No statistically significant associations were detected (Supplementary Table S2). The effect sizes for paternal anxiety (BAI) and depressive symptoms (BDI) measured in childhood, predicting the child’s pituitary structure and function, were small (*r*_p_ < 0.239). We also note that the observed power for detecting potential influences of paternal BDI on child pituitary volume and DHEA/DHEA-S were also small to medium, which suggests that we were underpowered to detect potential statistically significant associations. The observed power for detecting the associations between paternal BAI and child pituitary volume and DHEA/DHEA-S were however, medium to large (0.617–0.829), suggesting that if the effect sizes are reliable estimates of the true population effects, then we were adequately powered to detect such effects.

### 3.4 Indirect associations between paternal mental health measures and child behavioral outcomes via DHEA

**Figure 2:**
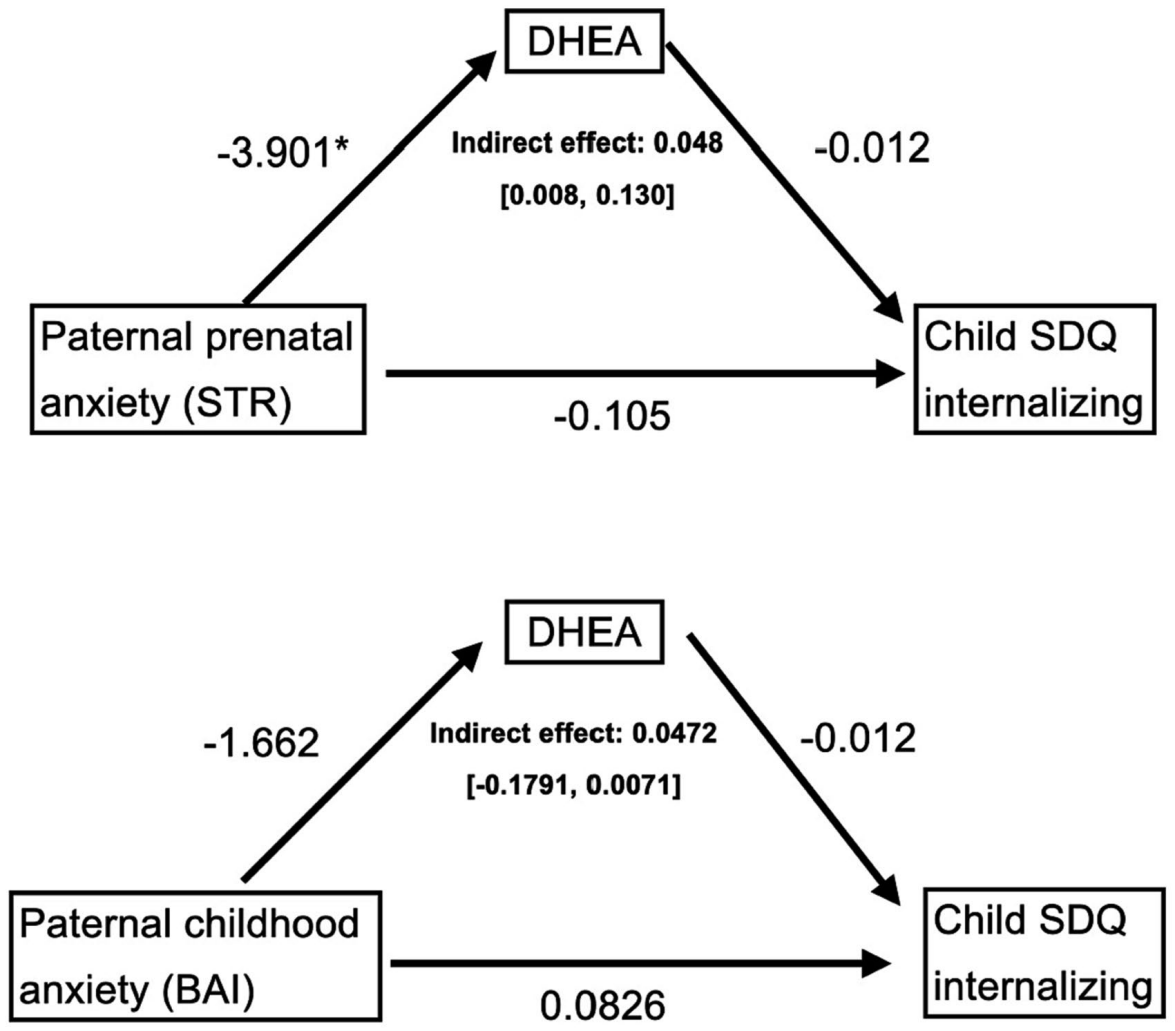
Mediation models testing the indirect associations between paternal anxiety, assessed during the prenatal period (top) and at the childhood assessment (bottom), and child internalizing symptoms at 6–8 years old via child DHEA levels. Control variables include paternal anxiety assessed in childhood (top) or prenatally (bottom), as well as maternal prenatal (STR) and childhood (BAI) anxiety, and sex, thus making the models comparable. Only the prenatal paternal anxiety (top) mediation model was significant. STR, prenatal anxiety questionnaire. BAI: Beck Anxiety Inventory; DHEA: dehydroepiandrosterone; STR: anxiety scale; SDQ: Strengths and Difficulties Questionnaire. *p < 0.05.

Indirect (mediation) analyses were conducted to test whether paternal prenatal anxiety symptoms (STR) were indirectly associated with behavioral (SDQ internalizing and externalizing symptoms) and cognitive outcomes (WISC scores) in the child via child DHEA levels; controlling for maternal prenatal anxiety (STR) and child sex. As shown in Figure 2, higher paternal prenatal anxiety symptoms (STR) were indirectly associated with higher internalizing symptoms in the child, via lower baseline DHEA levels in the child, over and above any effect of childhood paternal anxiety symptoms measured in childhood or prenatal/childhood maternal anxiety symptoms (indirect effect = 0.0482, CI = [0.0075, 0.1299]). This effect was specific to prenatal paternal anxiety symptoms (STR), as the model assessing anxiety symptoms in childhood (BAI) did not reach statistical significance [CI = −1791, 0.0071]. Moreover, this association was driven by the emotional subscale (indirect effect = 0.025, CI = [0.002, 0.073]). These statistically significant relationships were maintained when running sensitivity analyses that excluded the two participants with “high” DHEA-S levels. The models testing for externalizing and cognitive outcomes were not significant (data not shown).

## 4 Discussion

To our knowledge, this is the first study investigating paternal programming of stress, anxious, or depressive symptoms on their offspring’s pituitary structure and function, and their relationship with child cognitive-behavioral outcomes. We found that prenatal paternal self-reported anxiety symptoms were associated with child HPA function as measured by DHEA levels. Higher paternal anxiety symptoms during pregnancy were associated with lower DHEA levels 6–8 years later in their children, controlling for prenatal maternal anxiety symptoms. Although these findings are based on the 61 families recruited for the ancillary study from a larger longitudinal birth cohort, we note that this association had a consistent effect size (*r*_p_ ~ −0.3) in all models controlling for potential covariates, including maternal and paternal education and annual household income measured during pregnancy, as well as the time of day and season of salivary hormone sampling. In turn, using an indirect-effect analysis, we found that lower child DHEA levels were an intermediate factor explaining an indirect relationship between paternal prenatal anxiety symptoms and higher levels of internalizing symptoms (particularly emotional difficulties) in the child during middle childhood. Interestingly, findings appeared to be specific to paternal anxiety symptoms during the prenatal period, as opposed to the middle childhood period, given that only the prenatal anxiety model was significant. Moreover, post-hoc power analyses suggest that we were adequately powered to detect potential associations between concurrent paternal anxiety symptoms and child DHEA levels, if the sample effect size is a good estimate of the population effect size (observed power = 0.829; Supplementary Table S2). This finding is consistent with our hypothesis that mental health assessments during the prenatal period, as opposed to the childhood period, would be more strongly associated with child neuroendocrine outcomes, given that the neuroendocrine system development is programmed prenatally. These results suggest that paternal mental health may be an important modifiable risk factor for child mental health, and that paternal mental health should be given consideration for targeted interventions, as for mothers, as early as the prenatal period.

These findings highlight the importance, in the field of developmental neuroendocrinology, of: (1) obtaining biopsychosocial data on the family unit (mother–father-child triad); (2) measuring neuroendocrine intergenerational risk transmission in a prospective manner; (3) distinguishing paternal vs. maternal influences; and (4) identifying modifiable parental factors as early as possible, in order to optimize developmental trajectories.

We find evidence partially consistent with the organizational-activational hypothesis of neuroendocrine development. The first tenet posits that the intra-uterine environment, including exposures to psychological or physical stress, can result in lasting alterations in the organization and hence the function of the central nervous system (CNS) (Schulz et al., 2009). This early developmental process preferentially affects neuroendocrine brain structures such as the hypothalamic–pituitary axes, thereby resulting in what is commonly referred to as HPA and HPG “programming” (Rulli et al., 2018; Matthews and McGowan, 2019). The second tenet posits that the brain’s sensitivity to the organizational effects of steroid hormones decreases as the child matures, i.e., exposure to steroid hormones earlier in life would tend to have a more lasting impact on brain organization and structure while later developmental exposures would result in more reversible changes in brain function and activation. As such, prenatal and postnatal (enduring into childhood) exposures to symptoms of anxiety and depression via the parents can serve to differentially organize neural substrates, as well as impact the activation of various endocrine functions later in life (Schulz et al., 2009).

In relation to these frameworks, our study finds evidence to suggest that: (1) prenatal paternal factors can predict the child’s hormonal function during adrenarche and are distinct from that of maternal factors; and (2) prenatal paternal mental health factors may have stronger associations with the child’s hormonal function compared to postnatal (childhood) paternal mental health factors measured in middle childhood. In contrast, our data do not support stable or lasting paternal organizational effects detectable during middle childhood, given that we did not detect an association between prenatal paternal symptoms of stress, anxiety, or depression and pituitary structure as measured by its volume. This may be due to methodological limitations of structural MRI, as only macroscopic changes in pituitary structure can be detected by MRI. We thus cannot exclude whether paternal organizational effects occurred at a more microscopic, cellular, or molecular level. We also note that the effect size was small, and the observed power was also small to medium, possibly suggesting that our sample size might not have been sufficiently large to detect structural differences. Relatedly, we note that no sex difference was detected in pituitary volume, which we largely attribute to the age and pre-pubertal stage of the children, given that pituitary volume is reportedly larger in girls, beginning around age 10 and throughout adolescence due to HPG-axis hormones (Peper et al., 2010; Wong et al., 2014; Whittle et al., 2020), even after correction for total brain volume (Wong et al., 2014; Whittle et al., 2020). It is also possible that paternal mental health symptoms may be associated with other brain structures of the HPA axis. For example, hypothalamic nuclei or the zona reticularis of the adrenal cortex (via increased adrenocorticotropic hormone secretion stimulating activation of the 17,20 lyase enzyme, resulting in subsequent zona reticularis growth; Majzoub and Topor, 2018). Unfortunately, it was not possible to measure these neuroendocrine structures in our current sample, or, as far as we know, with existing structural MRI using a 3 T research scanner. Moreover, our single assessment of paternal mental health (during the first trimester) or more generally a history of mental health (as with the STR questionnaire) precluded our ability to consider the timing and potentially the fluctuating chronicity of paternal mental health symptoms during pregnancy. As it has been found for maternal mental health problems (Rees et al., 2019), we cannot rule out the possibility that chronic paternal mental health symptoms may have had a more important effect on child brain structure organization over time.

Our findings suggest the need to further consider a potential timing effect of the exposure to paternal mental health symptoms and its sequelae. The relation with timing is suggested by the specificity of the relationship between prenatal paternal anxiety symptoms and child internalizing symptoms, which were indirectly associated via child DHEA levels, in contrast to the concurrent/postnatal paternal anxiety symptoms, which was not (see Figure 2). We note that in a related study from the same sample (Jones et al., 2023b) associations between paternal mental health and child cognitive and behavioral outcomes were not explained by parental relationship quality or paternal self-perceived parenting measured in infancy. In the present study, prenatal paternal mental health was measured as early as possible during the 1^st^ trimester, and it is plausibly correlated with paternal mental health pre-conception. Thus, one potential mechanism for the association between paternal mental health symptoms and child neuroendocrine and behavioral outcomes may be related to direct biological pathways via sperm epigenetics (Lombó and Herráez, 2021). It has been shown that mental health status can alter sperm epigenetics, in turn influencing development of the offspring (Marcho et al., 2020), which may occur via paternal gene expression that programs placental development (Crespi, 2020; Dini et al., 2021). As such, one preconception pathway may occur via paternal gene expression which drives placental development, the maternal-fetal interface which is necessary for fetal growth and survival (Crespi, 2020; Dini et al., 2021). The field of genomic imprinting has revealed that sperm predominantly controls the growth and differentiation of extraembryonic tissues such as the placenta and yolk sac (Bhadsavle and Golding, 2022). Studies have consistently shown that preconception paternal exposures such as age (Denomme et al., 2020), obesity (Mitchell et al., 2017; Jazwiec et al., 2022), toxicants (Ding et al., 2018), cannabis (Innocenzi et al., 2019) and alcohol (De Cock et al., 2017; Thomas et al., 2022) result in sex-specific alterations of placental imprinted gene expression, as well as reduced placental weights and disruptions in placental histology. Importantly, the placenta is essential for regulating transport of nutrients and by-products, including maternal hormones between the fetal and maternal circulation, for protecting the fetus from the maternal immune system, and for producing hormones to support fetal development (Rossant and Cross, 2001; Adamson et al., 2002). Although direct evidence linking preconception paternal mental health factors and placental gene expression remains limited, both human and rodent models of intergenerational effects of trauma and chronic stress experience have reported epigenetic changes to sperm DNA methylation and subsequent associations with increased susceptibility to mental health conditions and disease risk in the offspring (Chan et al., 2018). Consistent with previous findings, one study in mice showed paternal preconception stress drives sex-specific transcriptional changes in the placenta, specifically tied to an upregulation in expression of gene sets involved in metabolic signaling and immunity in the placenta of female offspring (Cissé et al., 2020). Unfortunately, a preconception impact on the sperm epigenome could not be tested in our study since sperm were not collected from the fathers. The design of future birth cohort studies would benefit from collecting preconception data from the fathers to prospectively test whether mental health status influences sperm epigenetics, placental gene expression, the intra-uterine environment, and in turn influence child neuroendocrine and cognitive-behavioral development, building on other birth cohort studies and epigenetic inheritance (Pembrey and Team, 2004; Gapp et al., 2014; Fraser et al., 2016).

While existing literature suggests that higher DHEA levels during childhood and adolescence may support cognitive functions such as attention and working memory (Campbell, 2006; Nguyen et al., 2013, 2016, 2017; Nguyen, 2018; Witchel et al., 2020), there are also studies suggesting that DHEA is adversely associated with behavior, increasing the risk of developing both internalizing and externalizing symptoms (Mundy et al., 2015). These associations seem to be stronger in children with a predisposition for conduct and other externalizing disorders (Goodyer et al., 2003; Sontag-Padilla et al., 2012). Our findings further highlight the complexity of endocrine-behavioral relationships, as they indicate that any association between prenatal paternal mental health and DHEA levels in the offspring may preferentially be associated with internalizing (rather than externalizing) behaviors, in particular emotional problems during middle childhood.

Strengths of this study include the use of data from a longitudinal birth cohort, the 3D Study, and a prospective follow-up specifically designed to assess the potential associations between paternal factors on child neuroendocrine and cognitive-behavioral development. The 3D team collected biopsychosocial measures from mother–father-child triads during the prenatal and postnatal periods (up to 2 years) and encourages follow-up developmental studies on the cohort. In the context of our prospective follow-up study, when the children were aged 6–8 years, we collected maternal-paternal-child mental health data, as well as child salivary hormones, structural MRI, behavioral and cognitive outcomes. We evaluated predictive contributions of each parent while controlling for the other due to the availability of equivalent prenatal and childhood assessments of anxiety and depression. We also collected adrenarcheal hormones during a critical period of neural and social development, which is unique to humans and the great apes. We performed gold-standard manual segmentation of the PG from MRI, as well as parsed the anterior from the posterior PG, which is rarely done despite them being structurally and functionally distinct. In this way, we were able to assess individual relationships between pre- and postnatal (i.e., into middle childhood) paternal mental health, organizational (i.e., PG structure) and activational (i.e., hormone levels) measures, as well as cognitive and behavioral outcomes in the child. This allowed us to disentangle associations between parental mental health factors during the prenatal and childhood periods, and child neuroendocrine and behavioral development.

Limitations of our study include the small sample size, based on 61 children and their parents, and generalizability to vulnerable populations, rural and low socio-economic groups (Fraser et al., 2016). Moreover, mean parental self-reported scores of anxious and depressive symptoms for both mothers and fathers were low, indicating minimal presence of symptoms associated with mental health issues in parents participating in the paternal sub-study, and a more conservative test of our hypotheses. As such, our findings may not be generalizable to a population of parents experiencing clinical levels of anxiety or depression. Similarly, children in our sample scored within normal ranges on all SDQ subscales, including emotional symptoms (Shojaei et al., 2008), albeit this was a parental self-report measure. Thus, it is possible that fathers who reported higher mental health symptoms may have been more in-tune with these slightly higher, yet mild symptoms if they also provided the parental report of their child on the SDQ; particularly since the association seems to be driven by the emotional subscale. Unfortunately, we did not systematically collect data to determine which parent completed the SDQ, and could not account for this in our analyses. It is nonetheless interesting that even mild symptoms of prenatal paternal mental health may be associated with later child neuroendocrine and behavioral outcomes at subclinical levels, thus increasing the generalizability of our findings to subclinical populations. Future studies could address the associations between a greater range of symptoms of parental mental health and child outcomes, and should include assessment of current child stressors and family unit factors and the social environment as important factors to consider, as planned in the 3D transition study (Leblond et al., 2023). In addition, our findings could be due, at least in part, to the use of different instruments to measure paternal anxiety during the prenatal (STR) vs. concurrent childhood (BAI) periods. Still, both scales show a significant degree of overlap over several years in the self-reported items they measure, with STR screening for the presence/absence of symptoms suggestive of a clinically significant anxiety disorder as defined by DSM-V, whereas BAI measures levels of anxious symptomatology with a higher focus on somatic symptoms of stress. Regardless of this limitation, our results support screening for anxiety disorders in both parents as early as possible during pregnancy to prevent adverse developmental effects in the offspring. The results also suggest the potential added advantage of using a risk assessment for clinically significant signs of impairment and of social and occupational dysfunction due to anxiety (as in this study, the STR), rather than overall scores of self-reported levels of anxiety (BAI). Our findings suggest that future research should consider the inclusion of multiple parental assessments, including the preconception period, throughout pregnancy (i.e., prenatal - first, second, third trimester), and across childhood, while including varying components of both maternal and paternal mental health (e.g., stressors, anxiety, and depressive symptoms), and in particular, a screening measure for anxiety. Moreover, considering these factors in a single statistical model would make it possible to examine unique or compound effects of maternal and paternal mental health more comprehensively on child developmental outcomes (Black et al., 2019). Another limitation of this study is the lack of altered neuroendocrine function between the father and child across time. Addressing this in future studies would require collecting paternal sperm to conduct epigenetic analyses of relevant hormone promoter genes (e.g., methylation), at the time of conception and in the offspring cells (e.g., blood). In summary, our data highlight the importance of future birth cohort studies to gather pre-conception paternal data in addition to maternal data to assess potential biological pathways of child development more reliably, for example via sperm epigenetics.

## 5 Conclusion

From a sample of 61 children and their parents recruited as part of a larger longitudinal birth cohort study, this study found that prenatal paternal self-reported anxiety symptoms predicted lower salivary DHEA in children during adrenarche and that DHEA levels were an intermediate factor that accounted for associations between prenatal paternal anxiety symptoms and child internalizing symptoms. These data suggest that more research is needed to determine whether prenatal paternal factors could be important for offspring hormonal programming during a critical neuroendocrine event -adrenarche- and suggest an indirect association with increased child internalizing symptomatology. These data highlight the need for additional research between modifiable prenatal paternal factors and child developmental outcomes, and build a foundation for future studies to collect epigenetic measures in the father and child to address intergenerational transmission.

## Data availability statement

The raw data supporting the conclusions of this article will be made available by the authors, without undue reservation. The data are under the jurisdiction of the CHU Sainte-Justine Research Ethics Committee and subject to current provincial and national privacy laws guiding their ethical use in Québec. To submit a request, please visit http://www.irnpqeo.ca/en/researchers/ or contact the corresponding author.

## Ethics statement

The studies involving humans were approved by Research Institute of the McGill University Health Center Research Ethics Board and the CHU Ste-Justine Research Center Ethics Board. The studies were conducted in accordance with the local legislation and institutional requirements. Written informed consent for participation in this study was provided by the participants’ legal guardians/next of kin.

## Author contributions

T-VN, JRS, JT, SD, WF, CH, GM, SP, and NC-R conceived and designed the paternal study. SLJ and T-VN conceived this sub-analysis study and oversaw trainees. CC and JL performed paternal study data collection and data entry. SLJ processed all MRI images. SLJ and VB segmented the pituitary glands. SLJ, T-VN, and JL oversaw hormone analyses and data entry. GE managed the database with input from SLJ, T-VN, JL, and CC. GE performed imputations and statistical consultation. SLJ and VB performed statistical analyses. VB wrote the first draft of the manuscript in consultation with SLJ and T-VN. SLJ and TCM led the revisions of the article through to submission and publication. TCM oversees all ongoing aspects of the paternal study database. All authors contributed to the article and approved the final accepted version for publication version.

## Footnotes

^1^http://bic-mni.github.io
