# Supplementary material for: Prenatal paternal anxiety symptoms predict child DHEA levels and internalizing symptoms during adrenarche
Source: Front Behav Neurosci. 2024 Jan 4;17:1217846. doi: 10.3389/fnbeh.2023.1217846 (PMC10794355; doi:10.3389/fnbeh.2023.1217846)
Supplement: Supplementary file 1 [file Table_1.docx]

**Table S1.** Summary of non-significant linear regression analyses for prenatal variables predicting pituitary gland volume and hormone levels.

|  | ***R^2^*** | ***B*** | ***SE of B*** | ***β*** | *r_p_* | ***p*** | ***Observed power*** |
| --- | --- | --- | --- | --- | --- | --- | --- |
| **Prenatal Predictors** |  |  |  |  |  |  |  |
| PSS to Total PG Volume | .050 |  |  |  |  |  |  |
| Paternal PSS |  | .6.78 | 4.919 | .201 | .197 | .174 | .762 |
| Maternal PSS |  | -2.399 | 4.496 | -.078 | -.078 | .596 | .356 |
| Sex^a^ |  | 17.377 | 28.485 | .088 | .089 | .545 | .403 |
| PSS to Anterior PG Volume | .046 |  |  |  |  |  |  |
| Paternal PSS |  | 4.981 | 0.159 | .159 | .157 | .281 | .654 |
| Maternal PSS |  | -1.943 | 4.172 | -.068 | -.068 | .644 | .314 |
| Sex^a^ |  | 24.126 | 26.436 | .132 | .132 | .366 | .571 |
| PSS to Posterior PG Volume | .042 |  |  |  |  |  |  |
| Paternal PSS |  | 1.803 | 1.464 | .180 | .177 | .224 | .712 |
| Maternal PSS |  | -0.456 | 1.338 | -.050 | -.050 | .735 | .237 |
| Sex^a^ |  | -6.750 | 8.476 | -.115 | -.115 | 0.430 | .508 |
| PSS to DHEA Imputed | .038 |  |  |  |  |  |  |
| Paternal PSS |  | -2.231 | 2.816 | -.111 | -.110 | .432 | .488 |
| Maternal PSS |  | 3.025 | 2.582 | .165 | .162 | .247 | .669 |
| Sex^a^ |  | -9.861 | 15.751 | -.087 | -.087 | .534 | .394 |
| PSS to DHEA-S Imputed | .007 |  |  |  |  |  |  |
| Paternal PSS |  | -3.625 | 98.760 | -.005 | -.005 | .971 | .063 |
| Maternal PSS |  | -23.018 | 90.553 | -.036 | -.036 | .800 | .158 |
| Sex^a^ |  | -281.978 | 552.338 | -.072 | -.071 | .612 | .286 |
| CES-D to Total PG Volume | .069 |  |  |  |  |  |  |
| Paternal CES-D |  | 15.284 | 10.363 | .214 | .212 | .147 | .787 |
| Maternal CES-D |  | 0.851 | 2.750 | .045 | .046 | .758 | .216 |
| Sex^a^ |  | 26.663 | 28.279 | .135 | .138 | .351 | .583 |
| CES-D to Anterior PG Volume | .081 |  |  |  |  |  |  |
| Paternal CES-D |  | 14.481 | 9.549 | .219 | .218 | .136 | .575 |
| Maternal CES-D |  | 0.525 | 2.534 | .030 | .031 | .837 | .157 |
| Sex^a^ |  | 32.229 | 26.058 | .176 | .179 | .222 | .708 |
| CES-D to Posterior PG Volume | .014 |  |  |  |  |  |  |
| Paternal CES-D |  | 0.802 | 3.194 | .038 | .037 | .803 | .180 |
| Maternal CES-D |  | 0.327 | 0.848 | .058 | .057 | .702 | .262 |
| Sex^a^ |  | -5.567 | 8.716 | -.094 | -.094 | .526 | .416 |
| CES-D to DHEA Imputed | .015 |  |  |  |  |  |  |
| Paternal CES-D |  | -4.598 | 6.083 | -.108 | -.106 | .453 | .464 |
| Maternal CES-D |  | 0.508 | 1.594 | .046 | .045 | .751 | .212 |
| Sex^a^ |  | -6.675 | 16.052 | -.059 | -.059 | .679 | .270 |
| CES-D to DHEA-S Imputed | .022 |  |  |  |  |  |  |
| Paternal CES-D |  | 143.363 | 210.527 | .097 | .096 | .499 | .424 |
| Maternal CES-D |  | 26.024 | 55.168 | .068 | .067 | .639 | .304 |
| Sex^a^ |  | -308.483 | 555.550 | -.078 | -.078 | .581 | .350 |
| STR to Total PG Volume | .009 |  |  |  |  |  |  |
| Paternal STR | -.078 | 1.281 | 2.365 | .086 | .082 | .591 | .318 |
| Maternal STR |  | -0.944 | 2.921 | -.051 | -.049 | .748 | .201 |
| Sex^a^ |  | 9.077 | 27.295 | .051 | .051 | .741 | .208 |
| STR to Anterior PG Volume | .014 |  |  |  |  |  |  |
| Paternal STR |  | 1.051 | 2.159 | .077 | .051 | .629 | .208 |
| Maternal STR |  | -0.851 | 2.667 | -.050 | -.028 | .751 | .130 |
| Sex^a^ |  | 16.339 | 24.921 | .100 | .090 | .516 | .347 |
| STR to Posterior PG Volume | .017 |  |  |  |  |  |  |
| Paternal STR |  | 0.230 | 0.810 | .045 | .043 | .778 | .180 |
| Maternal STR |  | -0.093 | 1.000 | -.015 | -.014 | .926 | .088 |
| Sex^a^ |  | -7.262 | 9.346 | -.119 | -.118 | .441 | .446 |
| STR to DHEA-S Imputed | .035 |  |  |  |  |  |  |
| Paternal STR |  | -29.502 | 50.594 | -.087 | -.085 | .563 | .329 |
| Maternal STR |  | -39.140 | 59.900 | -.097 | -.095 | .517 | .365 |
| Sex^a^ |  | -526.780 | 573.253 | -.133 | -.133 | .363 | .496 |
|  |  |  |  |  |  |  |  |

Note: N=47-55. PSS: Perceived Stress Scale; CESD: Center for Epidemiological Studies Depression Scale; STR: anxiety scale; DHEA: dehydroepiandrosterone; DHEA-S: dehydroepiandrosterone-sulfate; PG: pituitary gland. ^a^0=boy, 1=girl, r_p_= partial correlation.
