# Supplementary material for: Prenatal paternal anxiety symptoms predict child DHEA levels and internalizing symptoms during adrenarche
Source: Front Behav Neurosci. 2024 Jan 4;17:1217846. doi: 10.3389/fnbeh.2023.1217846 (PMC10794355; doi:10.3389/fnbeh.2023.1217846)
Supplement: Supplementary file 2 [file Table_2.docx]

**Table S2***.* Summary of non-significant linear regression analyses for postnatal paternal mental health predicting concurrent child pituitary gland volumes and hormone levels.

|  | ***R^2^*** | ***B*** | ***SE of B*** | ***β*** | *r_p_* | ***p*** | ***Observed power*** |
| --- | --- | --- | --- | --- | --- | --- | --- |
| **Postnatal Predictors** |  |  |  |  |  |  |  |
| BAI to Total PG Volume | .077 |  |  |  |  |  |  |
| Paternal BAI |  | 4.339 | 3.952 | .186 | .158 | .278 | 0.617 |
| Maternal BAI |  | -1.747 | 2.313 | -.128 | -.110 | .454 | 0.454 |
| Sex^a^ |  | 47.470 | 27.165 | .247 | .247 | .087 | 0.825 |
| BAI to Anterior PG Volume | .135 |  |  |  |  |  |  |
| Paternal BAI |  | 6.143 | 3.646 | .277 | .239 | .099 | 0.812 |
| Maternal BAI |  | -2.688 | 2.133 | -.206 | -.181 | .214 | 0.683 |
| Sex^a^ |  | 56.086^*^ | 25.060 | .306 | .310 | .030 | .908 |
| BAI to Posterior PG Volume | .069 |  |  |  |  |  |  |
| Paternal BAI |  | -1.803 | 1.190 | -.258 | -.216 | .137 | 0.767 |
| Maternal BAI |  | 0.942 | 0.697 | .229 | .091 | .183 | 0.381 |
| Sex^a^ |  | -8.616 | 8.183 | -.149 | -.128 | .298 | .519 |
| BAI to DHEA Imputed | .053 |  |  |  |  |  |  |
| Paternal BAI |  | 3.615 | 2.149 | .265 | .229 | .099 | .829 |
| Maternal BAI |  | -1.039 | 1.353 | -.121 | -.107 | .446 | .476 |
| Sex^a^ |  | -2.105 | 15.864 | -.018 | -.019 | .895 | .113 |
| BAI to DHEA-S Imputed | .039 |  |  |  |  |  |  |
| Paternal BAI |  | 85.207 | 71.755 | .189 | .164 | .241 | .675 |
| Maternal BAI |  | -43.052 | 45.198 | -.151 | -.132 | .345 | .571 |
| Sex^a^ |  | -357.686 | 529.816 | -.093 | -.094 | .503 | .424 |
| BDI to Total PG Volume | .057 |  |  |  |  |  |  |
| Paternal BDI |  | -1.055 | 3.131 | -.048 | -.049 | .738 | .216 |
| Maternal BDI |  | 0.506 | 1.637 | .044 | .045 | .758 | .201 |
| Sex^a^ |  | 43.587 | 27.343 | .226 | .226 | .118 | .788 |
| BDI to Anterior PG Volume | .086 |  |  |  |  |  |  |
| Paternal BDI |  | -1.393 | 2.935 | -.067 | -.069 | .637 | .295 |
| Maternal BDI |  | .609 | 1.535 | .056 | .058 | .693 | .252 |
| Sex^a^ |  | 50.833 | 25.636 | .277 | .278 | .053 | .872 |
|  |  |  |  |  |  |  |  |
| BDI to Posterior PG Volume | 0.020 |  |  |  |  |  |  |
| Paternal BDI |  | 0.337 | 0.957 | .051 | .051 | .726 | .224 |
| Maternal BDI |  | -0.102 | .501 | -.030 | -.030 | .839 | .146 |
| Sex^a^ |  | -7.246 | 8.361 | -.125 | -.125 | .391 | .508 |
| BDI to DHEA Imputed | .048 |  |  |  |  |  |  |
| Paternal BDI |  | -2.020 | 1.788 | -.155 | -.138 | .264 | .592 |
| Maternal BDI |  | 1.212 | 0.981 | .170 | .152 | .222 | .638 |
| Sex^a^ |  | -3.331 | 15.904 | -.029 | -.025 | .835 | .136 |
| BDI to DHEA-S Imputed | .020 |  |  |  |  |  |  |
| Paternal BDI |  | -44.559 | 60.114 | -.103 | -.103 | .462 | .460 |
| Maternal BDI |  | -4.047 | 32.996 | -.017 | -.017 | .903 | .106 |
| Sex^a^ |  | -354.053 | 534.714 | -.092 | -.092 | .511 | .415 |
|  |  |  |  |  |  |  |  |

Note. N=51-55. BDI: Beck Depression Inventory; BAI: Beck Anxiety Inventory; DHEA: Dehydroepiandrosterone; DHEA-S: DHEA-sulphate; PG: pituitary gland. *p<0.05. ^a^ 0=boy, 1=girl, r_p_= partial correlation.
