# Supplementary material for: Prenatal paternal anxiety symptoms predict child DHEA levels and internalizing symptoms during adrenarche
Source: Front Behav Neurosci. 2024 Jan 4;17:1217846. doi: 10.3389/fnbeh.2023.1217846 (PMC10794355; doi:10.3389/fnbeh.2023.1217846)
Supplement: Supplementary file 3 [file Data_Sheet_1.pdf]

Supplementary Material

Prenatal Paternal Anxiety Symptoms Predict Child DHEA levels and Internalizing Symptoms during Adrenarche

Sherri Lee Jones*, Victoria De Braga, Christina Caccese, Jimin Lew, Guillaume Elgbeili, Natalie Castellanos-Ryan, Sophie Parent, Gina Muckle, Catherine M. Herba, William D. Fraser, Simon Ducharme, Julia Barnwell, Jacquetta Trasler, Jean R. Séguin, Tuong-Vi Nguyen, Tina Montreuil

*** Correspondence:** Sherri Lee JONES, PhD sherri.jones@affiliate.mcgill.ca

1.1 Supplementary Figures

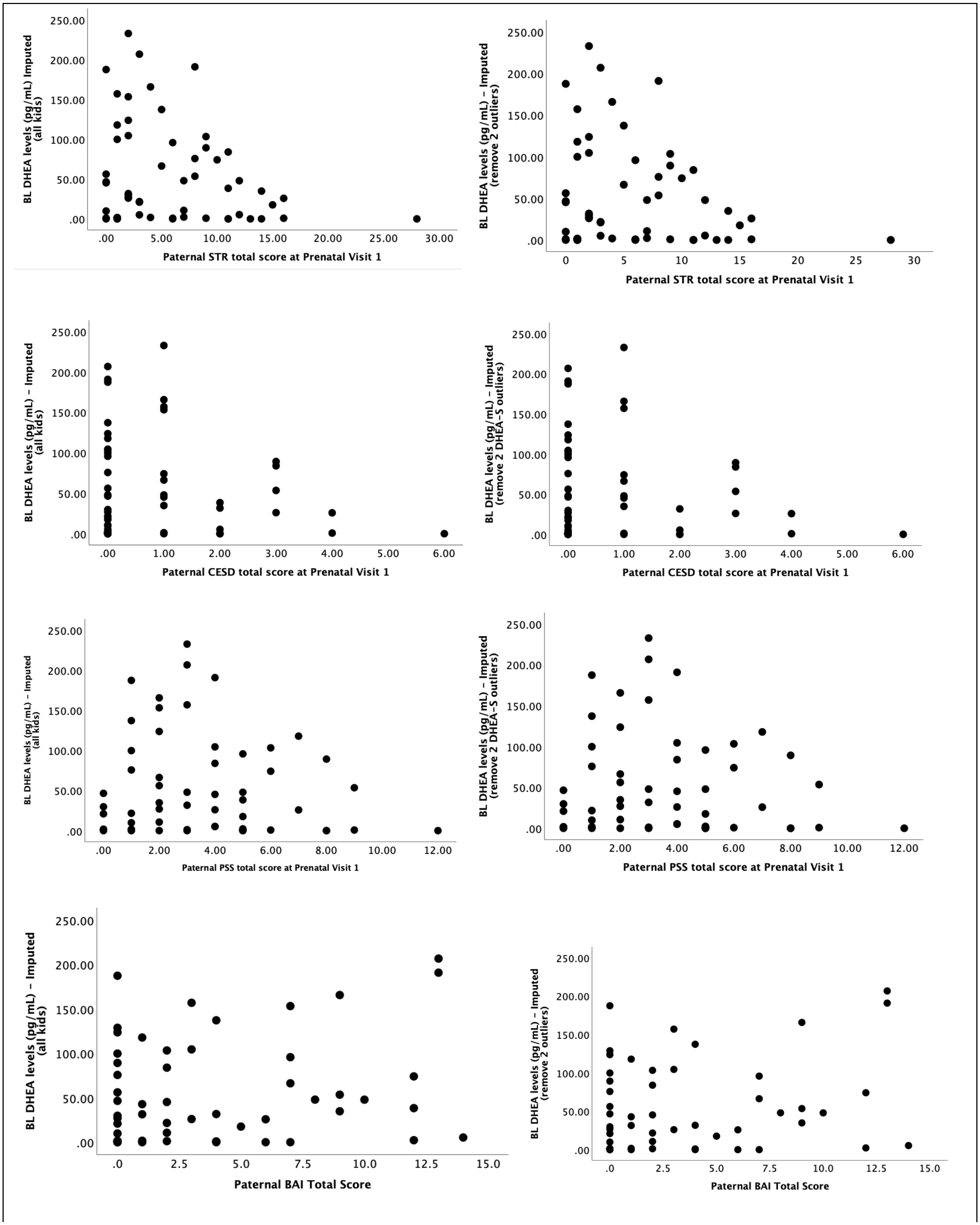
BL DHEA levels (pg/mL) Imputed (all kids)
250.00
200.00
150.00
100.00
50.00
.00
.00
5.00
10.00
15.00
20.00
25.00
30.00
Paternal STR total score at Prenatal Visit 1
BL DHEA levels (pg/mL) – Imputed (remove 2 outliers)
250.00
200.00
150.00
100.00
50.00
.00
0
5
10
15
20
25
30
Paternal STR total score at Prenatal Visit 1
BL DHEA levels (pg/mL) – Imputed (all kids)
250.00
200.00
150.00
100.00
50.00
.00
.00
1.00
2.00
3.00
4.00
5.00
6.00
Paternal CESD total score at Prenatal Visit 1
BL DHEA levels (pg/mL) – Imputed (remove 2 DHEA-S outliers)
250.00
200.00
150.00
100.00
50.00
.00
.00
1.00
2.00
3.00
4.00
5.00
6.00
Paternal CESD total score at Prenatal Visit 1
BL DHEA levels (pg/mL) – Imputed (all kids)
250.00
200.00
150.00
100.00
50.00
.00
.00
2.00
4.00
6.00
8.00
10.00
12.00
Paternal PSS total score at Prenatal Visit 1
BL DHEA levels (pg/mL) – Imputed (remove 2 DHEA-S outliers)
250.00
200.00
150.00
100.00
50.00
.00
.00
2.00
4.00
6.00
8.00
10.00
12.00
Paternal PSS total score at Prenatal Visit 1
BL DHEA levels (pg/mL) – Imputed (all kids)
250.00
200.00
150.00
100.00
50.00
.00
.0
2.5
5.0
7.5
10.0
12.5
15.0
Paternal BAI Total Score
BL DHEA levels (pg/mL) – Imputed (remove 2 outliers)
250.00
200.00
150.00
100.00
50.00
.00
.0
2.5
5.0
7.5
10.0
12.5
15.0
Paternal BAI Total Score

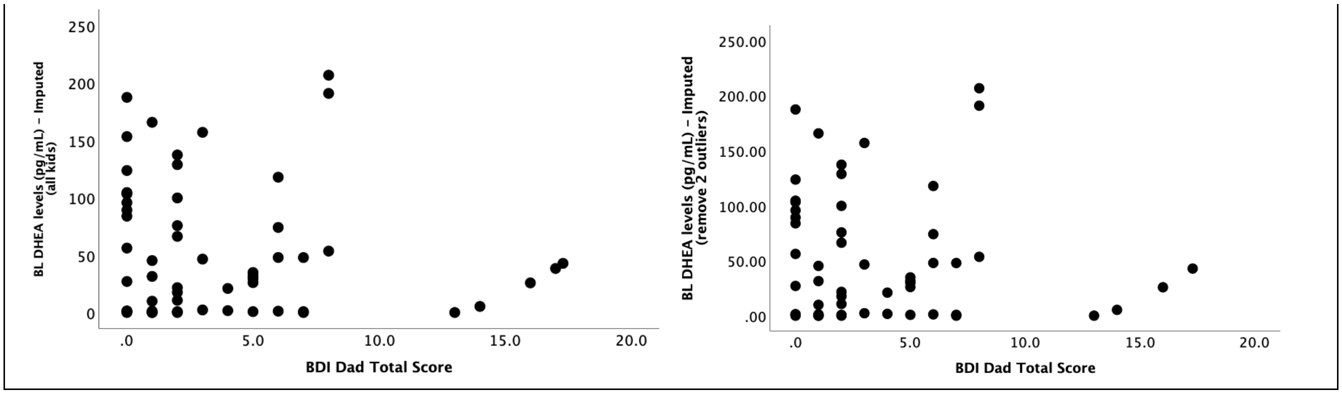

Supplementary Figure 1. Scatterplots showing bivariate correlations between child DHEA (dehydroepiandrosterone) levels at ages 6-8 and paternal mental health measures at the prenatal (STR, CES-D, PSS) or concurrent childhood (BAI, BDI) assessment. Correlations including the full sample (left panels) and after excluding the two DHEA-S outliers (right panels) are shown. CES-D, Center for Epidemiological Studies Depression Scale; PSS, Perceived Stress Scale; STR, Anxiety Scale; BAI: Beck Anxiety Inventory; BDI: Beck Depression Inventory.

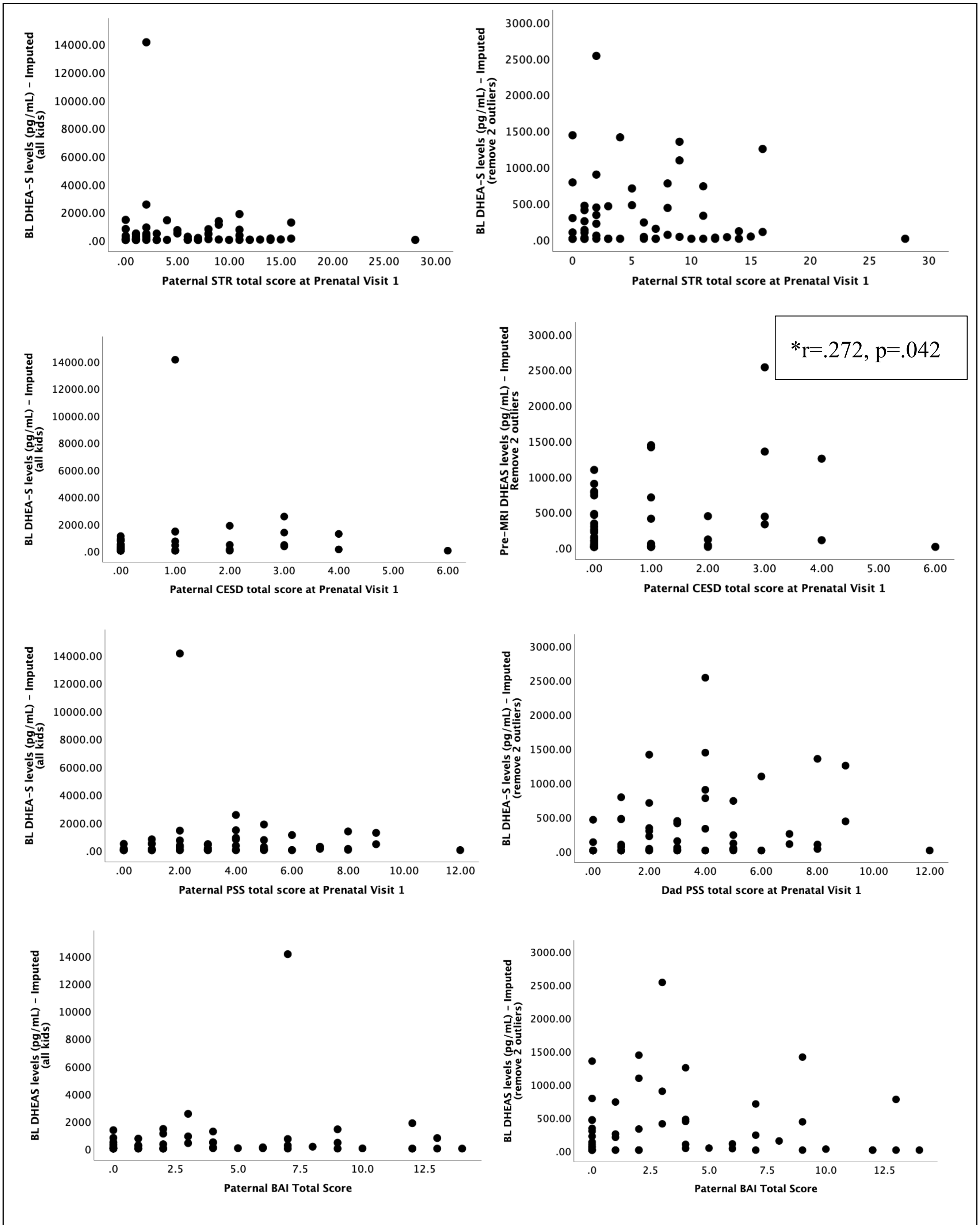
BL DHEA-S levels (pg/mL) – Imputed (all kids)
Paternal STR total score at Prenatal Visit 1
BL DHEA-S levels (pg/mL) – Imputed (remove 2 outliers)
Paternal STR total score at Prenatal Visit 1
BL DHEA-S levels (pg/mL) – Imputed (all kids)
Paternal CESD total score at Prenatal Visit 1
Pre-MRI DHEAS levels (pg/mL) – Imputed Remove 2 outliers
*r=.272, p=.042
Paternal CESD total score at Prenatal Visit 1
BL DHEA-S levels (pg/mL) – Imputed (all kids)
Paternal PSS total score at Prenatal Visit 1
BL DHEA-S levels (pg/mL) – Imputed (remove 2 outliers)
Dad PSS total score at Prenatal Visit 1
BL DHEAS levels (pg/mL) – Imputed (all kids)
Paternal BAI Total Score
BL DHEAS levels (pg/mL) – Imputed (remove 2 outliers)
Paternal BAI Total Score

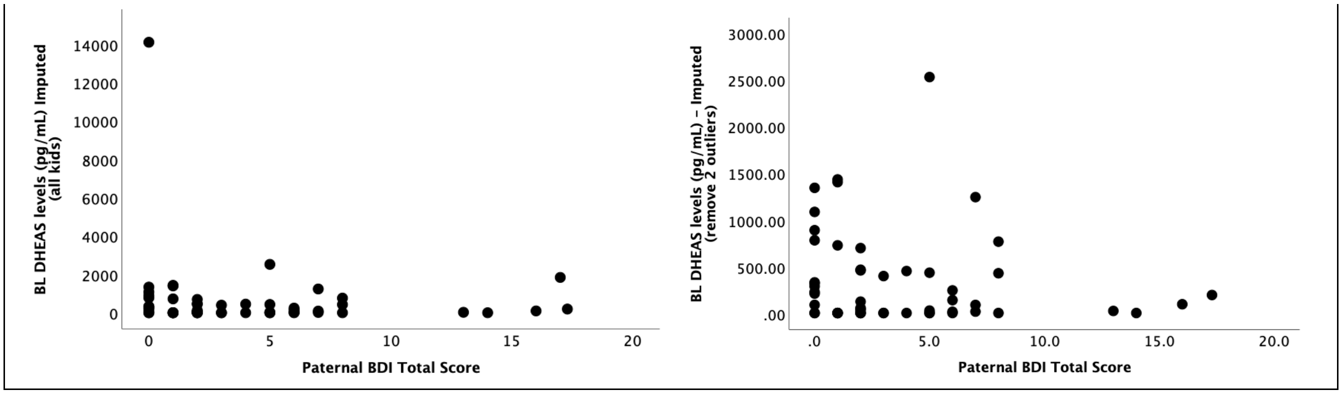

Supplementary Figure 2. Scatterplots showing bivariate correlations between child DHEA-S (dehydroepiandrosterone-sulfate) levels at ages 6-8 and paternal mental health measures at the prenatal (STR, CES-D, PSS) or concurrent childhood (BAI, BDI) assessment. Correlations including the full sample (left panels) and after excluding the two DHEA-S outliers (right panels) are shown. CES-D, Center for Epidemiological Studies Depression Scale; PSS, Perceived Stress Scale; STR, Anxiety Scale; BAI: Beck Anxiety Inventory; BDI: Beck Depression Inventory. *p<.05.
